# Preventing Malnutrition in Post-Conflict, Food Insecure Settings: A Case Study from South Sudan

**DOI:** 10.1371/currents.dis.54cd85fa3813b0471abc3ebef1038806

**Published:** 2014-07-07

**Authors:** Amy Paul, Shannon Doocy, Hannah Tappis, Sonya Funna Evelyn

**Affiliations:** Department of Health Policy and Management, Johns Hopkins School of Public Health, Baltimore, Maryland, USA; Department of International Health, Johns Hopkins School of Public Health, Baltimore, Maryland, USA; Department of International Health, Johns Hopkins School of Public Health, Baltimore, Maryland, USA; Adventist Development and Relief Association, Silver Spring, Maryland, USA

## Abstract

Background: Decades of civil conflict compound the challenges of food insecurity in South Sudan and contribute to persistent, high levels of child malnutrition. As efforts to prevent child malnutrition continue, there is a critical need for strategies that effectively supplement the diets of pregnant women and young children in transitional, highly food insecure settings like South Sudan. Methods: This mixed-methods case study of four communities in South Sudan reports on the diets of children under 2 years of age and explores household-level factors including household size, intrahousehold food allocation practices, and responses to scarcity that may have significant impact on the effectiveness of strategies relying on household ration distribution to supplement the diets of pregnant women and children under 2 years of age. Results: Participants reported experiencing increased scarcity as a result of prolonged drought and household sizes enlarged by the high volume of returning refugees. Although communities were receiving monthly household rations through a non-emergency food assistance program, most households had exhausted rations less than 30 days after receipt. Results showed that more than one half of children 12-17 months and one third of children 18-23 months consumed diets consisting of fewer than 4 food groups in the last week. Intrahousehold food allocation patterns give children first priority at meal times even in times of scarcity, yet adult women, including pregnant women, have last priority. Discussion: These findings suggest that distribution of supplementary household rations will likely be insufficient to effectively supplement the diets of young children and pregnant women in particular. In light of the multiple contextual challenges experienced by households in transitional, food-insecure settings, these findings support recommendations to take a context-specific approach to food assistance programming, in which considerations of intrahousehold food allocation patterns and broader cultural and environmental factors inform program design. Incorporating assessments of intrahousehold food allocation patterns as part of needs assessments for food assistance and voucher or cash transfer programs may contribute to more effective, context specific programming.

## Introduction

Despite decades of emergency food aid and, more recently, non-emergency food aid and development assistance programming in South Sudan, child malnutrition remains a persistent challenge. Data from the Sudan Household Health Survey in 2010 estimate that one in four children under five in South Sudan are stunted, one in five are wasted, and one in three are underweight.^1^ Further, measures at the household level show that nearly 17% of households are affected by stunting, 12% by wasting, and 14.3% by underweight.^1^ Decades of civil conflict and periods of prolonged drought compound the challenges of food assistance, and as conflict continues there is a growing need for effective food assistance strategies in transitional, highly food insecure contexts like South Sudan.

The recently concluded South Sudanese Health, Nutrition, and Empowerment (SSHiNE) program was the first multi-year assistance program (MYAP) in South Sudan. Implemented by the Adventist Development Relief Agency (ADRA), Concern Worldwide, Food for the Hungry, and Malaria Consortium, SSHiNE included supplementary ration provision following the “Prevention of Malnutrition in Children Under 2 Approach” (PM2A) in select areas of Northern Bahr-el-Gazal and Warrap states ****(Figure 1).****

**Map of Project and Assessment Areas:**
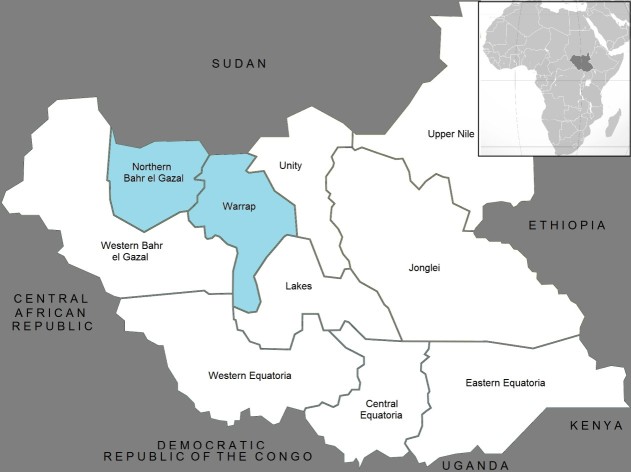
Assessment occurred in select areas of Warrap and Northern Bahr-el-Gazal States.

The PM2A strategy represents the most current strategy for preventing child malnutrition, and provides supplementary rations to all pregnant women and children under 2, regardless of nutrition status. Distributed as dry rations to be prepared at home, the PM2A promotes the distribution of individual rations to each pregnant women and child aged 6-23 months as well as an additional ration for other members of their household. The household ration is meant to prevent sharing of individual rations, incentivize program participation, and generally supplement household diets.^2^ While this strategy proved beneficial in its original evaluation in Haiti^3^ , the ability of household rations to effectively supplement the diets of pregnant women and children under two years of age is dependent on a number of household-level factors that vary across cultural and geographic settings, including underlying food insecurity, household size, intrahousehold food allocation patterns, and household response to scarcity.

South Sudan diverges from the typical setting of malnutrition prevention programs in a number of ways, including its history of political conflict and instability and its vulnerability to drought and acute shocks to food security. These macro level contextual factors may manifest at the household level in a number of ways, affecting household size, ability to produce food, and otherwise exerting influence over both the food available to the household and the practice of allocating food among household members. In addition to the influence of political and environmental context, little is known about normal patterns of intrahousehold food allocation patterns in households receiving food assistance, which is a long-time source of uncertainty for USAID Food For Peace programs.^4^ The academic literature on intrahousehold food allocation patterns remains fractured with a number of ethnographic studies from South Asia 5 ^,^ 6 ^,^ 7 but few rigorous studies from other regions. A recent review of intrahousehold food allocation literature found only 4 studies from Africa documenting food intake among members within the household and, further, wide variation in allocation patterns between and within geographic regions.^8^ Thus, for many food insecure settings, understanding local patterns of intrahousehold allocation will be an important step in the design of household rations, especially for programs that target particular members of the household, such as PM2A.

In order to better inform food assistance programming in post-conflict, highly food insecure settings like South Sudan, we sought to understand how the diets of individuals within the household are influenced by the political, environmental, and cultural context of South Sudan. This study triangulates data from household questionnaires, meal observations, and focus group discussions to report on the diets, demographics, and intrahousehold food allocation patterns of households in four communities in South Sudan, and how they may affect malnutrition prevention strategies relying on household ration distribution to supplement the diets of pregnant women and children under 2.

## Methods

**Study Population. **This mixed-methods case study reports on four communities in two states in South Sudan to understand the household context of participants in a malnutrition prevention food assistance program. For the purposes of this study, “household” is defined as anyone who eats meals and sleeps at the household compound. Communities in each state that were designated as PM2A areas (as compared to locations that were benefiting from other SSHiNE activities or where food distribution was not occurring) were selected for inclusion in the sampling frame based on security and access considerations and similarities to communities benefiting from the SSHiNE project. Of the communities in the sample, four communities (two in each state) were randomly selected for the final sample. The selected communities were in the payams of Kuach North (Gogrial West county) and Toch East, (Gogrial East county) in Warrap State and in Ariath (Aweil North county) and Gojuer Center (Aweil West county) in Northern Bahr el Ghazal State. Within each community, data collection included three approaches : meal observations, focus group discussions, and structured household questionnaires.

**Data Collection: **Data collection took place in January of 2012, six months after ration distribution began and within 30 days of the prior ration distribution, in order to ensure communities had been exposed to the ration intervention. The research team included a PI and two research assistants from Johns Hopkins University, along with a team of 6 bilingual data collectors (Dinka and English) who were SSHiNE staff members. All interviews and focus groups with community members were conducted in Dinka by the data collectors; data collectors were selected who did not have regular contact with project beneficiaries to reduce risk of reporting bias. Data collectors were trained on interview techniques, focus group facilitation and the data collection tools and participated in pilot testing and finalizing the household questionnaire, focus group discussion guides, and meal observation tool.

**Household questionnaires.** Six data collectors conducted a total of 80 household questionnaires. In each of the four communities, 20 adult female beneficiaries (pregnant women and/or mothers of children under two years of age) were interviewed using a structured questionnaire. Communities were segmented into “near” and “far” areas from the community market area to ensure geographic distribution, and mothers from each segment were sampled by convenience. Beneficiary status was assured by visual confirmation of ration card before beginning the questionnaire. The questionnaire incorporated validated and widely used instruments to assess household food security and diet quality, as well as household demographics. 10 ^,^ 11 ^,^ 12 Dietary data was collected for sorghum or millet, CSB, rice, bulgur, leafy vegetables, non-leafy vegetables (okra, pumpkin, tomato, cassava, sweet potato), eggs, milk, fish, beans, lentils, meat or poultry. For analysis, diets were assessed using a modified measure of dietary diversity, where foods were categorized into seven food groups (staples, leafy vegetables, non-leafy vegetables, milk, pulses, meat/fish, and eggs) and over a 7 day recall period to minimize the impact of day-to-day variation in food availability. Minimum dietary diversity was assessed according to WHO guidelines for assessing infant and young child feeding practices, which define minimum dietary diversity for children under two as consumption of food from at least four of the seven food groups listed above.^12^

Data from structured questionnaires were analyzed using descriptive statistics with STATA 11.

**Meal Observations. **Working in teams of two, the six data collectors completed a total of 16 focused meal observations among children under 2 years of age (4 in each community). In each study area, mothers of children under two and pregnant women were gathered by the village chief and program staff identified the first four women to volunteer to participate in a meal observation. Participation was determined in the morning the day of the meal observation in order to minimize potential for participants to alter meal preparations from usual practice. Participants in meal observations were in all cases different from those participating in focus groups. Observations documented the meal ingredients, preparation methods, and allocation of prepared foods, as well as the number of people served at each meal. A brief conversation with the meal preparer was conducted to understand how many people would eat the prepared food, including household members who were presently away but would return to eat later. Meal observations lasted from 45 minutes to approximately 2 hours, and included the period of preparation, allocation, and consumption by children only. Because adults ate often many hours after children, logistical constraints prevented observations from including consumption by adults, which would have occurred after dark.

**Focus Group Discussions. **Data collectors completed a total of 8 focus group discussions; 2 focus groups were conducted with 6-10 mothers of children under 2 years of in each of the 4 communities. Women were recruited by the village chief and asked to gather the morning of the focus group discussions, at which point they were divided into two groups. Working in teams of two, data collectors conducted focus groups in Dinka with one person facilitating while the second took notes. The facilitator followed a discussion guide on topics including household food security, intrahousehold food allocation, and allocation strategies in times of scarcity. Immediately following the focus group, the facilitator and note-taker wrote detailed responses to questions to ensure reliability and confirmability of results. Focus group data were analyzed using qualitative description and content analysis.^13^

Oral informed consent was obtained from each participant prior to initiation of the questionnaire, observation or focus group and beneficiary status was confirmed by possession of ration card. Permission to conduct the questionnaires, observations and focus groups was obtained from local authorities and the study was approved by the Institutional Review Board at the Johns Hopkins Bloomberg School of Public Health.

## Results

**Household Demographics.** Based on 80 structured household interviews, mean household size was 9.9 (SD 3.4), median size was 10 (range 5-20). Children under two years of age, the primary target group for PM2A programs were present in 73% of households; older children in the 2-4 and 5-17 year age groups were present in 83% and 96% of households respectively. The average household had 0.8 children <2, and 1.5 children 2-4 (total of 2.3 children <5 years of age), and 3.1 children 5-17 years of age with similar composition in terms of numbers of children within households in both states **(Table 1). **Households consumed an average of 1.6 meals per day with 38% of households consuming less than two meals per day.

**Table 1. Household Demographics:** 

**Household Size**	
Mean (SD)	9.9 (3.4)
Median (range)	10 (5-20)
**Children <2 years of age**	
Mean (SD)	0.8 (0.6)
Median (range)	1 (0,3)
% of households with children <2 years	73%
**Children 2-4 years of age**	
Mean (SD)	1.5 (1.2)
Median (range)	1 (0,7)
% of households with children 2-4 years	83
**Children 5-17**	
Mean (SD)	3.1 (2.0)
Median (range)	3 (0,8)
% of households with children 5-17 years	96%

**Diets of Children under 24 months of age.****Ninety-four percent of mothers of children 12-18 months of age, reported breastfeeding (median 5.5 times per day). Among mothers of children 18-23 months of age, 50% reported breastfeeding (median 1.5 times per day). Breast milk was a significant component of the diet, and by far the most frequently consumed food among 12-17 month olds, however, breast milk was supplemented with other food for both age groups. The most commonly consumed foods were staple cereals, with 72% of 12-17 month olds and 83% of 18-23 month olds consuming cereals in the last 7 days. Consumption of other food groups was less common than consumption of staple cereals, and large proportions children did not consume any foods from other food groups. Among 12-17 month olds, at least 50% of children had not consumed any foods from each of the remaining food groups of leafy or non-leafy vegetables, milk, eggs, fish, or pulses in the last week **(Figure 2)**.

**Food Group Consumption by Children 12-17 months of age:**
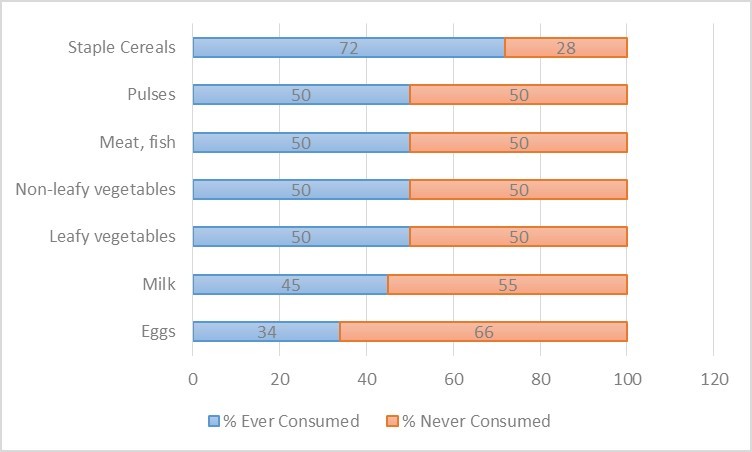
Food group consumption over last 7 days by children 12-17 months of age (n=18)

For 18-24 months old, the proportion of children not consuming any foods from groups other than staple cereals ranged from 26% (no consumption of meat or fish) to 53% (no consumption of eggs) **(Figure 3)**.

**Food Group Consumption by Children18-23 months of age:**
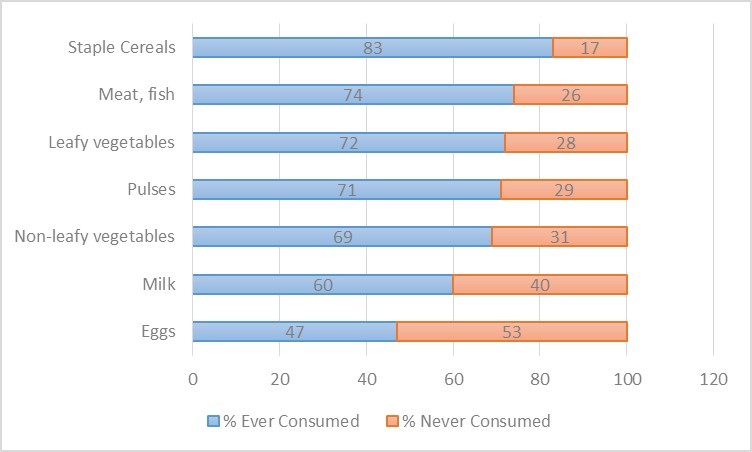
Food group consumption over last 7 days by children 18-23 months of age (n=36)

Over the last 7 days, 55% of children 2-17 months and 31% of children 18-23 months had consumed diets of below WHO recommended minimum dietary diversity cutoff of 4 food groups.

**Intrahousehold Food Allocation Patterns. **Women in focus groups described food allocation according to the age-mate system, where children of similar age eat together, sharing from the same dish. Food is allocated to young children first, then older children, then adult men, and lastly adult women. Children eat shortly after the meal is prepared, while adults often eat hours later, after dark. Food is allocated into dishes after preparation, and while children eat, the remaining plates are put aside for adults to eat later in the evening, after children go to sleep. During FGDs in all communities, participants emphasized that the study year was a time of particular scarcity because of extended drought conditions, which limited opportunities for household gardens and effectively turned the entire year into a “lean season” and resulted in frequent need to prioritize children and reduce their own portion sizes. Women additionally commented that absence of men from households contributed to scarcity because men were able to earn more income than women, which had consequences for the ability to buy food from the market to supplement household food stores in lean times. Based on FGDs, when the amount of food is inadequate, children are prioritized and adults skip meals or forego eating. In one community, men and youths ate together, and women shared food with the very young children, still prioritizing children before taking a portion for herself. Mothers reported that in times of scarcity, they will leave the portion for the children unchanged as much as possible, and reduce the portion she would normally save for women of the household, then, if necessary reduce the portion for men, and as last resort reduce the portion allocated for children. While blended foods such as CSB was not prepared in any meal observations, women in focus groups maintained that, when they had received CSB in the past, it was reserved for children and served on a shared plate to the youngest children.

**Household Meals. **Meal observations supported the dietary data collected in household questionnaires and consistently showed a limited variety of foods consumed. Most households had no ration foods remaining at the time of study; the exception were two households with a small amount of bulgur, which exhausted and combined with other grains to make enough for the meal. Most commonly, meals consisted of two dishes: a grain porridge and a soup of okra, ground peanuts (if available), dried fish (if available) and a local ingredient called “kombo,” which is a liquid made by straining water over the ash of a particular wood. In rare cases, where households had no other food available, the meal consisted entirely of a boiled soup made from the leaves of a wild plant called “akuor” which is considered a famine food.^14^

For nearly all households, the staple food was sorghum, but three households had maize or bulgur. Children were fed mostly the same food as adults. No households were observed to have stores of fruit or other snack foods; households near the market occasionally had a few packages of biscuits of small box of juice for children to snack on, but these were not observed to be regularly present in households. After meal preparation, the mother or oldest daughter allocated porridge into dishes according to the age-mate system described in focus groups: food was served first to the young children, followed by older children, while plates were set aside for adult men and what was left was set aside for adult women. There was no observable difference in portion size or serving order between male and female children of similar age, nor was this reported in focus groups. Only in one observation did the mother sit with the children to ensure the youngest child ate enough.

## Discussion

Designing and implementing household ration distribution strategies in a way that maximizes their effectiveness is critical given the limited availability of food aid resources. While targeting ration distribution to vulnerable populations is potentially a high-impact strategy, these findings raise significant challenges to the feasibility and effectiveness of providing individual and household rations in post-conflict, highly food insecure settings like South Sudan.

At the time of our assessment, less than one month after distribution of household rations, few of the households observed had any ration remaining; approximately 38% of households ate less than two meals a day and many were relying on famine foods. More than half of children 12-17 months and nearly one third of children 18-23 months consumed diets comprised of fewer than 4 food groups in the last week. Data on household food insecurity and coping mechanisms in this community, reported elsewhere,^15^ confirm that despite ration receipt, these communities remained highly food insecure.

Further, our findings indicate that households in South Sudan experience a confluence of contextual pressures that will present substantial obstacles to preventing malnutrition through the distribution of supplementary household rations. The absence of observed food stores and frequent mention in focus groups of a “lean year” as opposed to the lean “season” are indicative of the underlying food insecurity in the region, and the inability of households to obtain surplus food stores to protect from the shocks of drought or other acute threats. The likelihood of acute food shocks against a background of high food insecurity makes these household particularly vulnerable, and suggests that households may have few reliable food sources, which increases the likelihood that household rations provided through any food assistance programming will be a primary rather than supplementary source of food.

Secondly, the on-going political instability in the region affects households in a number of ways. Although the relative stability of in the region at the time of program initiation made it a candidate for non-emergency programming, it is also associated with high volumes of refugee return. Between October 2010 and January 2011, over 25,000 returnees are reported to have returned to Warrap and nearly 40,000 to Northern Bahr el Ghazal.^16^ The high volume of returnees in the study communities resulted in particularly high household sizes, increasing household energy requirements. The high household size observed in study communities may be a reflection of this influx, with mean household sizes of 10 and some as high as 20. In the case of PM2A and other preventive approaches, the household ration is meant to supplement the household food supply to prevent sharing of the rations intended for pregnant women and children under 2, yet it may not be feasible to distribute rations sufficient to meet the household energy gap in post-conflict, food insecure settings like South Sudan. Additionally, women in focus groups also reported that the absence of men from the household was detrimental to food security, because men could earn more money and thus provide more means to purchase food during times of scarcity. Thus, conflict resulted in both both the absence of men who were directly involved in conflict and the later return of displaced persons in high number, both mounting challenges to the household ability to meet its food requirements.

Finally, the practices of intrahousehold food allocation play a critical role in the distribution of the scare food available within the household. Our findings show that allocating a full portion to children first is the priority at meal times, even during periods of scarcity, while adult women are the first to experience reduced portions, and often skip meals in times of scarcity. While the priority given to children is encouraging and suggests household rations have potential to effectively supplement the diets of young children, our findings also present a number of challenges. The culture of shared-plate feeding of children of similar age means that children slightly above the target age range of food assistance strategies targeting children under two may also share in a portion intended for one child, and there are few mechanisms to ensure a specific child consumes the intended ration portion. Further, the lack of priority given to adult women, including pregnant women, suggests that pregnant women are most vulnerable to inadequate food intake in times of scarcity. Focus group discussions consistently identified the portion of food allocated for women as the first to be reduced in times of scarcity, and subject to the greatest reduction. Other studies of the Dinka in South Sudan also indicate that women of the household will always eat last, even if pregnant or breastfeeding, and that there is a tendency for women to give larger quantities of food to men while serving themselves smaller and often inadequate portions.^17^ Especially when occurring in the context of large household size and high food insecurity, intrahousehold food allocation patterns are likely to exacerbate the challenge of effectively supplementing the diets of pregnant women.

These findings suggest that additional or alternative strategies that more fully address the multiple contextual challenges experienced by households in South Sudan will be necessary to supplement the diets of pregnant women and young children. One potential strategy is integrating food assistance with behavior change, community agriculture and livelihoods training in order to simultaneously address the broader contextual challenges contributing to food insecurity. While the PM2A strategy does include such additional components, the experience of these communities suggests that integrated approaches may similarly face substantial barriers in effective implementation. For example, community agriculture will do little to assist households in a drought year; behavior change components require regular participation that may be difficult to achieve where beneficiaries have multiple competing demands on their time. While approaches that condition ration receipt on participation in behavior change activities or other complementary program components are a potential alternative, in highly resource constrained settings, the necessary monitoring of conditional approaches raise questions about opportunity costs associated with implementation. While a holistic approach to improving food security is needed, more research is needed to understand the most effective strategies to balance the long-term strategies to prevent malnutrition with the acute need of populations in post-conflict, food insecure settings, where contextual challenges may present significant barriers to complementary components of integrated programming.

More broadly, this works adds to a growing body of research highlighting the complexity and significance of intrahousehold food allocation patterns in preventing malnutrition. While the specific pattern of prioritizing children and deprioritizing adult women is supported by other work with Dinka communities, its contrast to patterns in other regions is consistent with claims that intrahousehold food allocation is highly context-specific, varying not only across regions,^18^^,^ 19 ^,^ 20 but across income and social class as well.^21^^,^ 22 Taken together, this work suggests that intrahousehold food allocation is a complex practice that can have significant implications for household-level programming, and supports recommendations for a highly context-specific approach to food assistance programming.^23^^,^ 24 Specifically, incorporating assessments of intrahousehold food allocation patterns into needs assessment for food assistance programming, as well as in voucher and cash transfer programs that may occur in food insecure settings, could be an important step in the design of more effective, context-specific program approaches.

This study carries several limitations. First, these data are derived from a rapid assessment of a small, non-representative sample and may not capture the full range of experiences of SSHiNE beneficiaries. In particular, recruiting participants for observations and focus-groups through the village chief may have resulted in participants being more vulnerable if they expected showing up would result in receiving some form of compensation. This limitation, however, does not change the informative value of understanding intrahousehold food allocation patterns and the reality that some portion of program beneficiaries were experiencing high levels of scarcity despite receipt of household rations. Overall, these data contribute to the small body of data available for South Sudan and present informative findings for the development of context-specific food aid programming for post-conflict, food insecure settings.
